# Basal and Stress-Induced Network Activity in the Adrenal Medulla *In Vivo*

**DOI:** 10.3389/fendo.2022.875865

**Published:** 2022-06-20

**Authors:** Jose R. Lopez Ruiz, Stephen A. Ernst, Ronald W. Holz, Edward L. Stuenkel

**Affiliations:** ^1^ Department of Molecular & Integrative Physiology, University of Michigan Medical School, Ann Arbor, MI, United States; ^2^ Department of Cell and Developmental Biology, University of Michigan Medical School, Ann Arbor, MI, United States; ^3^ Department of Pharmacology, University of Michigan Medical School, Ann Arbor, MI, United States; ^4^ College of Health and Life Sciences, Hamad Bin Khalifa University, Doha, Qatar

**Keywords:** adrenal medulla, stress response, chromaffin cell, *in-vivo*, electrophysiology

## Abstract

The adrenal medulla plays a critical role in mammalian homeostasis and the stress response. It is populated by clustered chromaffin cells that secrete epinephrine or norepinephrine along with peptides into the bloodstream affecting distant target organs. Despite been heavily studied, the central control of adrenal medulla and *in-situ* spatiotemporal responsiveness remains poorly understood. For this work, we continuously monitored the electrical activity of individual adrenomedullary chromaffin cells in the living anesthetized rat using multielectrode arrays. We measured the chromaffin cell activity under basal and physiological stress conditions and characterized the functional micro-architecture of the adrenal medulla. Under basal conditions, chromaffin cells fired action potentials with frequencies between ~0.2 and 4 Hz. Activity was almost completely driven by sympathetic inputs coming through the splanchnic nerve. Chromaffin cells were organized into independent local networks in which cells fired in a specific order, with latencies from hundreds of microseconds to a few milliseconds. Electrical stimulation of the splanchnic nerve evoked almost exactly the same spatiotemporal firing patterns that occurred spontaneously. Hypoglycemic stress, induced by insulin administration resulted in increased activity of a subset of the chromaffin cells. In contrast, respiratory arrest induced by lethal anesthesia resulted in an increase in the activity of virtually all chromaffin cells before cessation of all activity. These results suggest a stressor-specific activation of adrenomedullary chromaffin cell networks and revealed a surprisingly complex electrical organization that likely reflects the dynamic nature of the adrenal medulla’s neuroendocrine output during basal conditions and during different types of physiological stress.

## Summary

Prolonged and dysregulated stress to extrinsic and physiological challenges often drives many chronic diseases. The better understanding of the sympatho-adrenal stress response will impact and improve the treatment of several stress related illnesses. This work focusses on the study of the functional architecture of the adrenal medulla, a key component in neuronal stress response.

## Introduction

The adrenal medulla has long been recognized as playing a critical role in mammalian homeostasis and the stress response. Numerous projections from the central nervous system regulate the activity of the preganglionic neurons, which innervate individual adrenal medullary chromaffin cells, thereby regulating catecholamine and peptide secretion into the circulation.

The current study focuses on chromaffin cell electrical activity and networks (highly correlated, sequential, and reproducible firing of distinct cells) in living anesthetized rats.

Chromaffin cells have been extensively studied *in vitro*, both in cell culture and in tissue slices, to determine biochemical, cell biological and biophysical aspects of cell function, however these studies do not address the physiology of chromaffin cells in intact adrenal medulla. Studies in tissue culture have illuminated biochemical and physiological pathways that lead to exocytotic release of catecholamines and proteins. Electrophysiological studies of cells in adrenal medullary slices have revealed that cells in their natural tissue environment have faster release kinetics (1) and have variable degrees of electrical coupling (2–6). Fast-scan cyclic voltammetry and amperometry detect individual epinephrine and norepinephrine fusion events in cultured chromaffin cells (7, 8) and in slices of mouse adrenal medulla (9).

Numerous studies have investigated biochemical and physiological function of chromaffin cells in intact preparations. Indeed, there is a rich history of *in vivo* investigations of biochemical changes in adrenal medullary content upon secretion following intense stress and of *in vitro* studies of secretion of catecholamines and proteins from perfused adrenal medulla [for reviews see (10, 11); also (12)]. These studies established exocytosis as the mechanism of secretion in neuroendocrine tissue. More recently, an *ex vivo* preparation demonstrated spatially- and frequency- dependent epinephrine and norepinephrine secretion in the rat adrenal gland that is relevant to the stress response (13). An *in vivo* study measured extracellular potentials from chromaffin cells in response to splanchnic nerve stimulation and suggested a role of electrical coupling in enhancing catecholamine secretion (14).

In the present study we extended the investigation of adrenal medullary function *in vivo.* We have utilized silicon multielectrode array probes to record action potentials extracellularly that are generated by chromaffin cells upon electrical or stress-induce splanchnic nerve stimulation in anesthetized rats. We adapted signal processing techniques, which have been developed for elucidating central nervous system neuronal activation patterns, that enabled detection of simultaneous activity from as many as 40 chromaffin cells. There was a surprising richness of responses with spatially overlapping cell networks displaying distinctive electrical and physiological responses. The results suggest that rather than all chromaffin cells responding identically, there is a significant amount of specificity and tuning of chromaffin responses to physiological stimulation. To our knowledge, these experiments are the first to use multi-electrode arrays *in vivo* to examine the electrical and functional architecture of any endocrine gland.

## Materials and Methods

All animal handling and experimental procedures associated with or performed in this study followed National Institutes of Health (NIH) animal use guidelines and were approved by the Institutional Animal Care & Use Committee (IACUC) at University of Michigan (Approval Number PRO00009446). In addition, all research performed in this study complied with the Institutional Biosafety Committee at University of Michigan (Approval Number IBCA 00000592).

### Animals and Surgery

**Figure 1:**
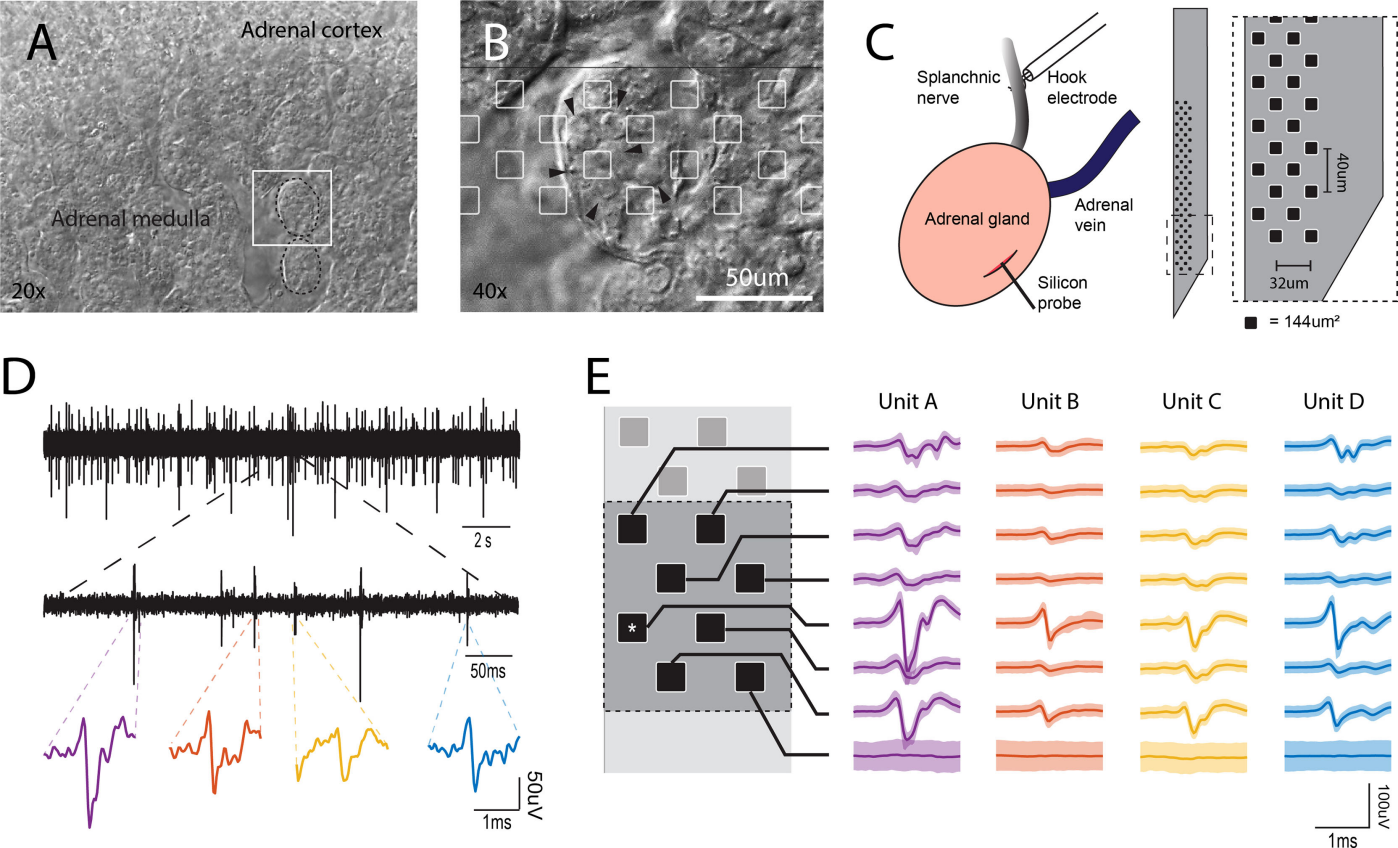
*In vivo* electrophysiological recordings from the intact adrenal medulla. **(A)** Low power bright field micrograph showing the adrenal cortex and the adrenal medulla. The white square represents the area shown in panel **(B)** B. Bright field micrograph showing the clustered architecture of the adrenal medulla (arrowhead = individual chromaffin cell). The silicon probe layout is shown in scale with the tissue, recording sites are presented as white squares. **(C)** Left, preparation in the anesthetized rat for simultaneous stimulation to the splanchnic nerve and the recording of the electrical activity in the adrenal medulla. Right, 64 channel silicon probe layout covering an area of 48x620 um (0.02976 mm^2^), the area of each individual recording site is 144µm^2^. **(D)** Spontaneous extracellular action potentials. Shown are recordings at low, medium and high expanded time scales. The most expanded recordings (bottom traces) show the waveforms of individual events. **(E)** A cell’s action potential projects distinct waveforms depending on its position to each recording site in the neighborhood (the asterisk represents the channel shown in **(D)**, the set of these slightly different waveforms define a unit’s template and it is unique for each cell (solid line ± 2SD).

All the experiments were carried out in female and male Sprague Dawley rats between 30 and 150 days old, housed in an enriched environment in a 12 hr. light–12 hr. dark cycle and climate‐controlled room (22°C) with free access to water and food. On the day of experimentation, subjects were transferred to an anesthetic induction chamber and the isoflurane anesthetic applied *via* a low flow vaporizer (SomnoFlo, Kent Scientific). Immediately after, the subject was transferred into a platform with temperature control set to 37C inside a Faraday’s cage to minimize external noise sources. Anesthesia was confirmed repeatedly during experiments by testing absence of a foot pain reflex. Surgery to reveal the adrenal gland was performed by making an incision through the left abdominal wall. Excess adipose tissue surrounding the adrenal gland was carefully removed. The adrenal gland was then immobilized to avoid recording artifacts associated with breathing or tissue movements and for prolonged stability of electrode placement by gently raising the adrenal and fixing placement with ring tip forceps attached to a micromanipulator. Electrical stimulation of the intact splanchnic nerve was performed using a bipolar platinum hook electrode placed around the nerve. Multi-site silicon electrodes (64 recording site M1 probe, Cambridge NeuroTech) were introduced under hydraulic micromanipulator control (Narashige) through a small incision made into the gland’s external capsule, which allowed stable recordings of multi-site electrical activity for extended periods (**Figure 1C**).

### Electrophysiological Recordings

All the experiments were carried out with the acute version of Cambridge Neurotech’s M1 probe (**Figure 1C**), paired with two 32 channel Intan RHD2132 headstages connected to the Open Ephys hardware and software (15). Prior to each experiment, the electrode impedance to a 1KHz sine wave was measured and recording sites with impedance > 200KΩ were excluded from the analysis. During the experiments, the 64 channels were simultaneously recorded at 30 ksamples/s and filtered from 300 to 6000 Hz with a digital 2nd-order Butterworth filter. Finally, the signal was common average referenced and stored for offline analysis. Immediately after the conclusion of experiments, probes were cleaned by dipping in an enzymatic detergent solution overnight, rinsed with distilled water the next morning and stored for further experiments.

### Electrical Stimulation

Pulse trains of 10 single phase 100 µs constant current pulses at 1-40 Hz were generated by triggering a current stimulus isolation unit with a TTL signal from a PulsePal Gen2 (Sanworks) controlled through the Open Ephys graphical user interface (GUI). Current intensity was increased from 0.0 to 4.5 mA in 0.5 mA steps following delivery of 5 pulse trains at each intensity.

### Spike Sorting and Analysis

Spike sorting was performed offline using Kilosort2 scripts and manually cured in Phy (16). A unit was classified as “good” when it displayed a constant firing rate during the basal period and few or no refractory period violations. After curating, homemade scripts were used to process and identify spikes and their timing. A unit’s template was obtained by extracting and averaging a ±3ms time window for every spike that corresponds to that specific unit from each channel in the original recording (waveform).

The firing rate was calculated by counting the number of events a unit had in 10 s bins. Changes in the firing rate of individual units were sometimes determined by calculating z-scores, where z = (x – μ)/σ. x represents the discrete firing rate of a unit, μ is the mean firing rate of the named unit during the basal period and σ is the standard deviation of the basal firing rate of that unit. A z-score greater than +2 or less than -2 indicates at statistically significant (p<0.05) increase or decrease respectively, compared to the baseline.

The correlation index between units was calculated by dividing the number of times a unit fired within ±10 ms of the occurrence of the reference unit. This was analyzed throughout the total number of spikes the reference unit presented during an analysis period.

### Pharmacology

The voltage-gated sodium channel antagonist tetrodotoxin (TTX) was locally applied by soaking a small cotton ball into a 10µM TTX solution and placing it onto the exposed splanchnic nerve. The nicotinic receptor antagonist mecamylamine (5 mg/kg, Sigma-Aldrich: M9020) was systemically administered with an intraperitoneal injection. Hypoglycemia was induced with three increasing intraperitoneal human insulin doses spaced by 15 minutes (10, 100 and 1000 µg/kg, Sigma-Aldrich: I9278), during this procedure blood glucose was periodically measured with an Accu-Chek meter (Roche) using blood samples obtained from the tail vein.

## Results

The adrenal medulla is populated by electrically excitable chromaffin cells that are organized into visually identifiable clusters (**Figures 1A, B** **)**. The cells are approximately spherical and are innervated by the sympathetic nervous system *via* the splanchnic nerve that emanates from the spinal cord. Neuronal tracing studies suggest multiple neuronal circuits originating from the brain stem and cerebral cortex that could influence chromaffin cell electrical activity *via* the splanchnic nerve (17, 18) and, thereby, provide control of specific cells or cell clusters upon different physiological stressors.

To investigate the electrical activity of chromaffin cells within the adrenal medulla of a living animal, we positioned a 64-channel silicon multi-electrode array that was guided through the capsule and the adrenal cortex into the medulla (**Figure 1C**). The recorded signals displayed multiple abrupt downward voltage deflections or spikes with no evident firing pattern (**Figure 1D**). The size (generally 40 μV -100 μV) and duration of the deflections indicate that the signals reflect action potentials from excitable cells rather than nerve fibers. This interpretation is based upon numerous studies of extracellular recordings in the CNS. For example, *in vivo* juxtacellular recordings in the CNS correlated the extracellular potential changes with the position of an electrode in relationship to cell bodies and axons (19). Because of the much higher current density from an excitable cell compared to an axon, the current source for an action potential detected by an extracellular electrode within 10-20 μm of a cell body is almost entirely the soma. There is virtually no contribution from nerve fibers that are within microns of the cell (19, 20).

Because of the multielectrode array architecture (**Figure 1B**), when a cell fires an action potential, the electrical signal can be recorded simultaneously in multiple recording sites. The waveforms are usually different for the different electrodes because of their unique positions with respect to the cellular source. A template is the resulting set of waveforms from a group of neighboring recording sites. It is specific for an individual cell. Since the position of each electrode in the array is fixed, a template is stable over time. If there are multiple cells very close to the original cell, each one will project a different set of waveforms to the same recording sites resulting in different templates (**Figure 1E**) with each distinct template identifying the firing of a specific cell. These considerations underlie template matching, the sorting technique used to interpret voltage changes in multiple electrodes in order to identify the firing of individual cells and their temporal interactions (see Methods). During a single experiment, template matching enabled the detection of multiple cells (average 17 cells) and in some cases greater than 40.

### Spontaneous Electrical Activity

**Figure 2:**
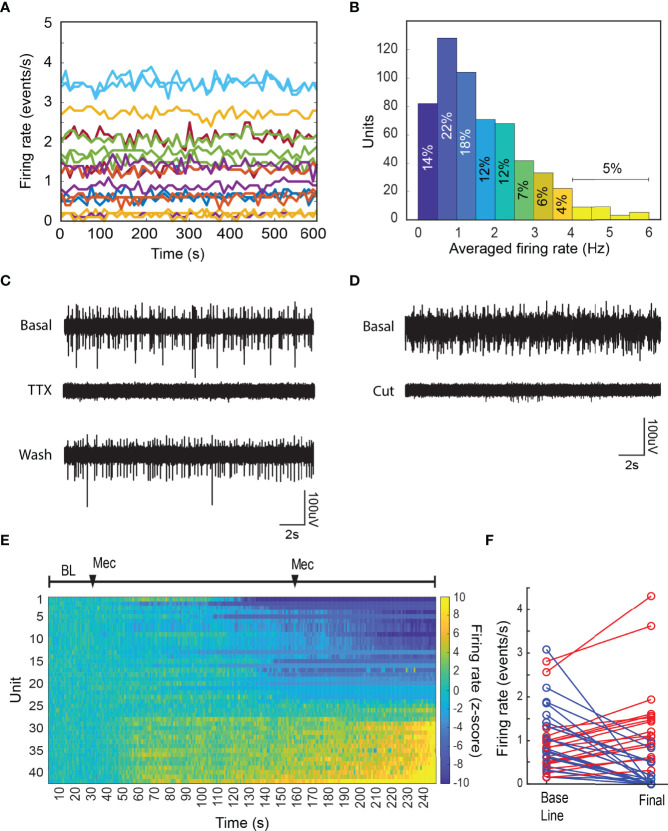
Basal chromaffin cell activity is driven by the splanchnic nerve. **(A)** Firing rate in 10 s bins from a set of units recorded for 10 minutes under basal conditions in a single preparation. **(B)** Averaged firing rate distribution from 576 units recorded during 40 preparations. **(C)** The local application of the sodium channel blocker TTX, reversibly blocks the activity in the adrenal medulla. The effect was seen in all recording sites. **(D)** Severing the splanchnic nerve proximally to the adrenal gland, irreversibly abolishes the electrical activity in the adrenal medulla. Basal activity was monitored for ten minutes prior to the cut, and the shown cut recording was done within a minute. **(E)** The systemic application of the nicotinic antagonist mecamylamine hydrochloride inhibits a subset of the chromaffin cells population in the adrenal medulla. The firing rate was estimated in 10 s bins and the normalized firing rate (z-score, see Methods) was calculated by considering the first 5 minutes of the recording as baseline. After the baseline period two 2 mg intraperitoneal doses of mecamylamine were administered at 5 and 25 minutes. **(F)** Shows the averaged firing rate from each unit during the baseline period vs. the final 5 minutes of the recording (blue=inhibited, red=increased).

Chromaffin cells fired in the absence of physiological stress (**Figure 2A**). The firing rates of individual cells varied widely, with ninety-five percent of the cells having firing rates between 0.2 and 4 Hz (550 out of 576), with a median of 1.36 Hz (**Figure 2B**). The baseline activity is consistent with the presence of circulating epinephrine even in the absence of stress. We found no significant differences between females and males in baseline firing rate for the studied age range (**Supplementary Figure 1A**). Interestingly, both females and males displayed an exponential decay in their firing rate when correlated to age (**Supplementary Figures 1B, C**).

We investigated the basis of the spontaneous activity. Application of the Na^+^ channel blocker tetrodotoxin (TTX) to the exposed nerve, reversibly inhibited the electrical activity, often reducing to zero the firing rate of individual chromaffin cells (**Figure 2C**). Activity partially recovered following TTX removal and local washing of the splanchnic nerve (**Figure 2D**). Cutting the splanchnic nerve caused an almost complete cessation of well-defined spiking activity. We conclude that under these conditions, most of the spontaneous activity in chromaffin cells is driven by basal levels of activation through the splanchnic nerve.

Rarely, low amplitude electrical activity was observed following disruption of splanchnic nerve function (**Figure 2D**). The sorting algorithm was unable to identify discrete sources for the residual activity that was likely unmasked by the disruption. The activity could reflect intrinsic spontaneous activity as has been reported *in vitro* (21, 22) from distant cells.

A major excitatory neurotransmitter released from the splanchnic nerve terminals is acetylcholine, which interacts with the nicotinic cholinergic receptors on the plasma membrane of chromaffin cells. The systemic administration of the nicotinic receptor antagonist mecamylamine (Mec) reduced the firing rate of many but not all chromaffin cells (**Figures 2E, F**). Unexpectedly, the firing rate of some cells increased, suggesting a compensatory, non-nicotinic cholinergic pathway for chromaffin cell activation and/or sensitivity of specific central nervous afferent controls to mecamylamine. In either case this indicates specific and potentially differential control of networks of chromaffin cells in the adrenal medulla.

### Network Activity in the Adrenal Medulla

**Figure 3:**
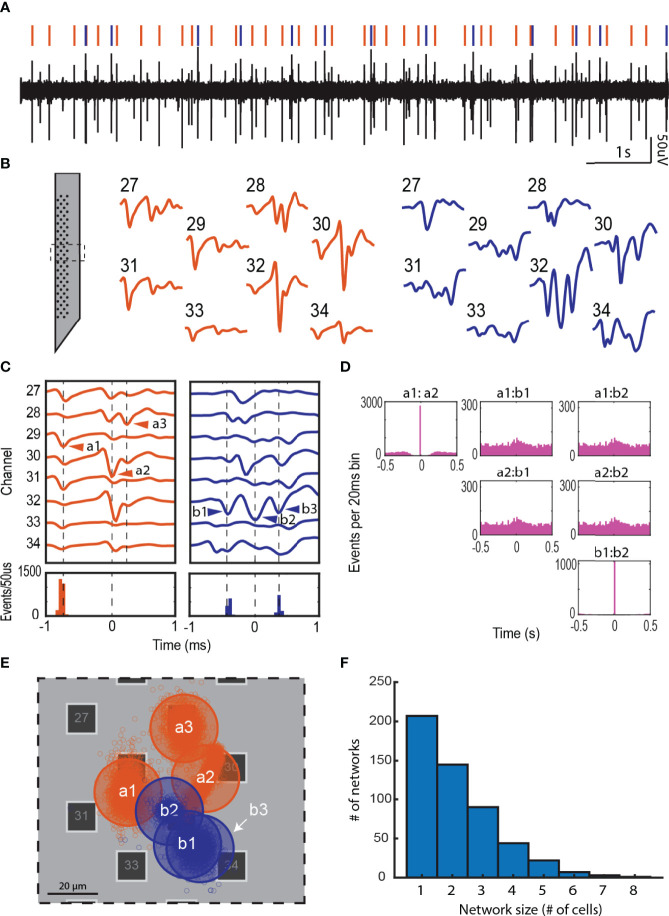
Local network activity in the adrenal medulla. **(A)** Single channel recording showing multiple spikes (downward deflections). **(B)** Waveforms from multiple electrodes from the experiment in panel A were sorted to derive two distinct templates shown in orange and blue. The corresponding waveforms in the single channel recording in panel A are indicated by orange and blue vertical lines. The templates displayed complex waveforms comprised of multiple spikes occurring in fixed succession. **(C)** Some of these spikes were identified as units on their own by the sorting algorithm (e.g. a1 and a2, and b1, b2 and b3), and others were manually identified from other templates derived from another unit (e.g. a3). A temporal histogram aligned to the reference unit in the template (time = 0 ms), shows a precise firing pattern of the units (a3 was excluded from this analysis). **(D)** A lower time resolution temporal histogram (20 ms per bin) shows a 1 to 1 correlation between a1:a2 and b1:b2, but no correlation between a and b (the total number of events for each unit are: a1 = 2818, a2 = 2875, b1 = 1061 and b2 = 1061). **(E)** Shows the approximate location of the cells associated with each network in **(C)** The small empty circles represent the result of the triangulation calculation for each action potential the cell fired. Transparent large circles represent 20 µm diameter cells centered at the centroids of the clouds of points associated with a particular unit. **(F)** Network size distribution from a total of 40 preparations.

Complex waveforms were frequently recorded at individual electrodes **(** **Figure 3A** **)**. The templates derived from these recordings displayed spikes occurring in fast succession across a neighborhood of recording sites (*e.g.* orange and blue templates in **Figures 3B, C**). The sorting algorithm was able to resolve these complex waveforms into individual units (*e.g.*, cells) that fire in a specific sequence with very precise latencies (*e.g.* a1→a2; b1→b2→b3 in **Figure 3C**). The fact that the successive spikes occurred well within an action potential refractory period of individual cells (≤ 1msec) and project distinct waveforms into the neighboring channels suggest distinct cellular sources of each spike on the complex waveforms **(** **Figure 3C** **)**. In addition, spikes with smaller amplitude were sometimes evident in the template (*e.g.* a3 in **Figure 3C**). These spikes were most likely generated by distant cells. Networks varied in size, from 1 to 8 cells **(** **Figure 3F** **)**.

Units in the same template (e*.g.*, a1 and a2 or b1 and b2) displayed a high temporal correlation (>90%, a1:a2 and b1:b2 in **Figure 3D**) indicating strongly coupled components in a single network. Despite being recorded at the same recording sites, units from different templates were completely uncorrelated (a1:b1, a1:b2, a2:b1 and a2:b2 in **Figure 3D**). By estimating the two-dimensional coordinates for each unit (cell), we found that the units in the different templates could be within tens of microns of one another **(** **Figure 3E** **)**. Thus, independent cellular networks can be spatially overlapping.

**Figure 4:**
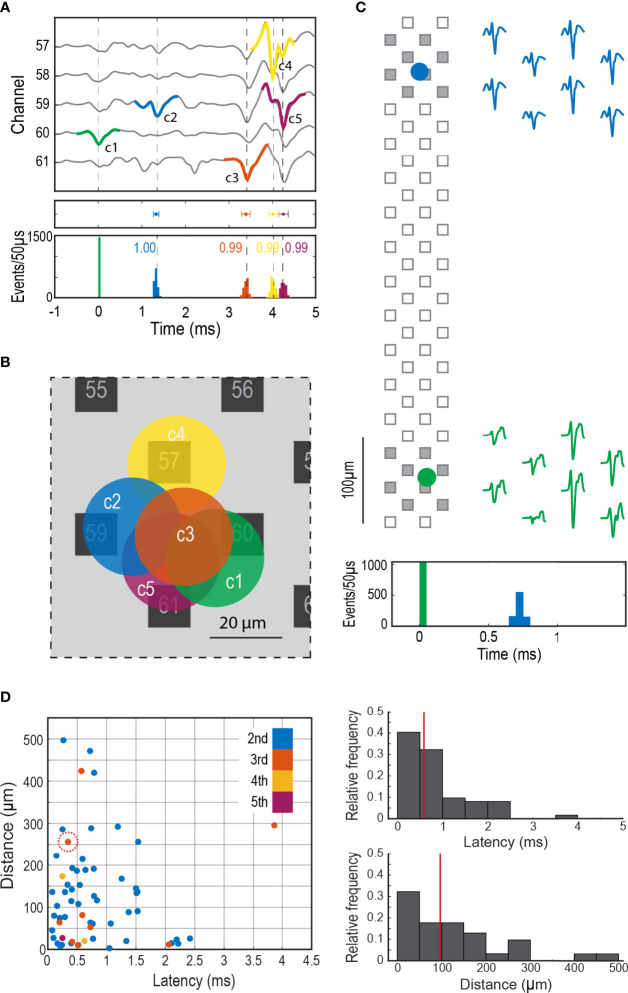
Synchronized firing in the adrenal medulla. **(A)** Firing sequence of a group of clustered cells, Top panel shows an extended template of the first unit (c1 in green) displaying the firing sequence of the later units (c2 to c5, blue, orange, yellow and purple), middle panel shows the mean latency ± SD (mean ± 2SD, c2 = 1.33 ± 0.06 ms, c3 = 3.39 ± 0.10 ms, c4 = 4.01 ± 0.10 ms and c5 = 4.26 ± 0.11 ms) in reference to c1, bottom panel shows the histograms in 50 µs bins of the firing distribution between the first cell and the subsequent cells in the network. **(B)** Shows the approximate location of the synchronized cells in A, the circles represent a 20 µm in diameter cell. **(C)** Firing synchronization between two distant cells, the circles represent the theoretical location of each cell over the probe, the filled squares correspond to the recording sites shown in the 2 ms templates on the right. The histogram at the bottom shows the firing distribution in 50 µs bins of the second cell (blue) in relationship to the first cell (green). **(D)** Left panel shows the relationship between latency and distance in coupled cells. The different colors indicate the order of appearance of each cellular component in the firing sequence. The abscissa for each dot is the latency from the previous action potential in the sequence; the ordinate is the distance between the calculated positions for the two cells. For example, the circled dot represents the 3^rd^ component of a firing sequence. In this case this cell fired approximately 0.4 ms after the second cell in that sequence and the calculated distance between this cell and its predecessor in the sequence was ~250 µm. The most frequent sequence had action potentials from only two cells (blue dots). Top right histogram shows the relative frequency distribution of the latency between consecutive cell pairs (n=62, the color code matches previous panels), bottom right histogram shows the relative frequency of the distance between cell pairs. The red vertical lines mark the median, 0.59 ms for latency and 97 µm for distance.

Latencies between cells in the same network occurred over a wide time frame, from hundreds of microseconds to a few milliseconds (**Figures 4A, D** **)**. Correlation indices between members of a network was usually close to 1 (**Figure 4A** **, bottom graph**), indicating that coupling rarely failed. There was a wide range of distances between cells in the same network (**Figure 4D** **)**, from as small as a cell diameter (20 μm, examples in **Figure 4B**) to as large as 500 μm (example in **Figure 4C**). The finding that networks can be distributed over hundreds of microns suggests that they can be comprised of cells across different cell clusters.

### Evoked Activity in the Adrenal Medulla

**Figure 5:**
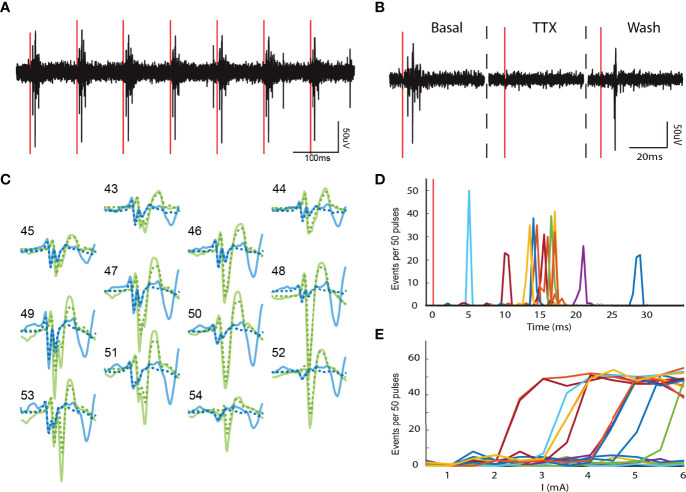
Electrically evoked chromaffin cell activity. **(A)** Electrical stimulation of the splanchnic nerve evokes activity in the adrenal medulla. Representative recording of a single phase 10Hz-3.5mA stimulation train (red vertical lines). **(B)** The local application of TTX to the splanchnic nerve reversibly blocked the evoked action potentials in the adrenal medulla. **(C)** Evoked templates (solid lines) from two units. The evoked templates are identical to their spontaneous counterparts (dotted lines). **(D)** The evoked units present distinct latencies that range from 5 to 30 ms after the stimulus (red vertical line). The data are from the same experiments as in panel **(C)**. **(E)** Distinct networks have different stimulus thresholds. The stimulus intensity was gradually increased from 0 to 6 mA in 0.5 mA steps.

To further investigate electrical parameters of chromaffin cell networks, we electrically stimulated the splanchnic nerve with different intensities (0 to 4500 µA in 500 µA steps). We found that splanchnic nerve activation evoked action potentials from chromaffin cells throughout an applied frequency spectrum of 1 Hz to 40 Hz (**Figure 5A**). Application of TTX locally to the nerve reversibly inhibited evoked activity (**Figure 5B**), thus again confirming the necessity of nerve excitability to drive chromaffin cell electrical firing. Notably, splanchnic nerve stimulation resulted in the similar complex waveforms as were recorded spontaneously at the same electrodes during the basal period (solid vs. dotted lines in **Figure 5C**). Thus, the same cellular network is activated for evoked and basal nerve activity. The evoked units fired at fixed latencies after splanchnic nerve stimulation that ranged from 5 to 30 ms **(** **Figure 5D** **)**. The firing probability of an entire network, not just individual units in the network, increased with higher stimulus intensities (**Figure 5E**), suggesting a robust reliability of the coupling.

Sometimes an extra spike in the waveform was identified by template matching upon evoked stimulation (**Figure 5C** **, blue solid trace**). The extra spike could reflect simultaneous activation of a fiber innervating a cell outside the network but adjacent to electrodes.

### Physiological Stress Response to Insulin-Induced Hypoglycemia

**Figure 6:**
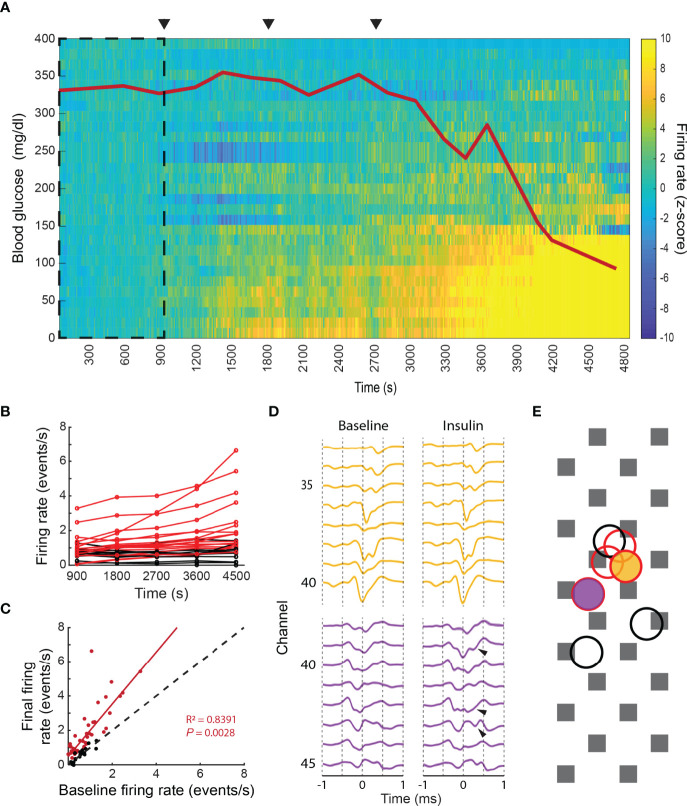
Physiological stress response in the intact adrenal medulla. **(A)** The intraperitoneal administration of 3 increasing doses of insulin, 10, 100 and 1000 µg/kg (arrowheads), induced a decrease in the blood glucose levels (red line) that correlated with a significant increase in the firing frequency of a subpopulation of chromaffin cells. The z-score was calculated per unit in 10s bins by taking the first 900s as baseline (dashed square). The experiment was divided into five 15 minutes periods: baseline (1-900s), 10 µg/kg (901-1800s), 100 µg/kg (1801-2700s), 1000 µg/kg (2701-3600s) and final (3601-4500s) **(B)** Individual averaged firing rate for the different periods (red indicates units whose final averaged firing rate increased by more than 2 times its baseline’s SD or averaged z-score above 2, black indicates the units that presented no change between its baseline firing rate and final firing rate or an averaged z-score between ±2). **(C)** Linear regression between the baseline firing rate vs. final firing rate for 64 units recorded from 3 preparations. The coefficients for the linear fitting are 1.523 (1.265, 1.78 with 95% confidence bounds), the 45-degree dashed line represent no change between the two evaluated periods. **(D)** Waveform changes during hypoglycemia. Top template (yellow) displayed no changes between the baseline and the 1000 µg/kg periods, whilst additional spikes (arrows) were found in the bottom unit in comparison to its baseline template, both units presented a statistically significant increase in their firing rate between the two periods. **(E)** Firing rate change does not correlate with the spatial localization of the cells. The yellow and purple filled circles indicate the localization of the units shown in **(D)**, which had increased firing rates upon hypoglycemia. The red and black unfilled circles represent the localization of additional units. Red circles represent cells whose firing rate increased; black circles represent cells whose firing rate decreased.

Our ability to record chromaffin cell activity in living animals allowed us to assess the physiological response of the adrenal medulla to a stressor. Anesthetized rats were challenged with increasing doses of insulin (10, 100 and 1000 mg/kg of i.p. insulin application) to initiate a hypoglycemic stress response. The challenge provoked a graded decrease in the blood glucose (red line in **Figure 6A**) and a sympathetic nervous system response that induces activation of the splanchnic nerve (23–25) and stimulation of the adrenal medulla. Correlated with the fall in blood glucose was a significant increase in the activity of a subset of chromaffin cells (**Figures 6A, B**). These cells displayed a 1.5x increase to their basal firing rate (95% confidence between 1.3 and 1.8 with r^2 =^ 0.84 and *p*=0.003, **Figure 6C**), suggesting a coordinated activation of a subpopulation of chromaffin cells. The ability to respond to hypoglycemia was not determined by spatial localization since networks that did and did not respond sometimes mapped to overlapping sites (black vs. red circles in **Figure 6E**). Occasionally, waveforms changed during the hypoglycemic challenge, displaying additional voltage deflections or spikes (**Figure 6D**). The changes may reflect the recruitment of neighboring cells to the network through enhanced neuronal coupling or increased gap junction coupling (see Discussion). These changes did not occur in waveforms whose frequency was unchanged during hypoglycemia.

**Figure 7:**
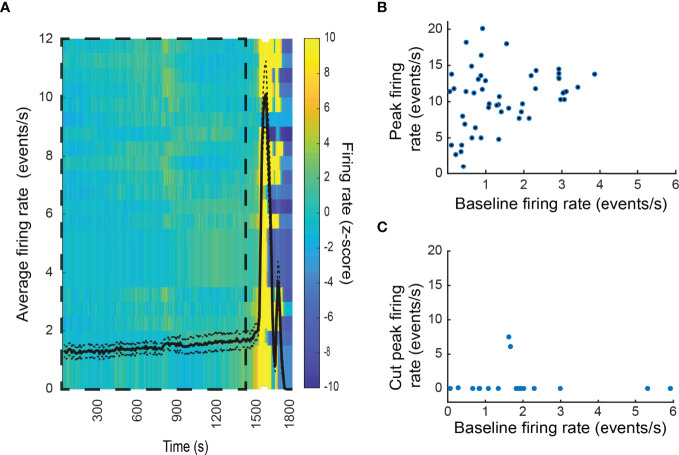
Physiological stress response to respiratory arrest in the intact adrenal medulla. **(A)** Generalized increase in the firing rate of the chromaffin cells was observed at the moment of respiratory arrest due to anesthesia overdose. Shown are the results of an individual experiment. Firing rates from 25 cells were analyzed. The black line corresponds to the average firing rate over time ± SEM (left axis). A normalized firing rate (Z-score) over time for each unit (row) is indicated by the color code (right axis). The baseline period is indicated by the dashed box. **(B)** Relation between the baseline firing rate and the peak firing rate after respiratory arrest. The combined results from three experiment are presented. The frequency 47 of 48 units increased following respiratory arrest. **(C)** Denervation virtually abolished the increase in firing rate upon respiratory arrest.

In contrast to the varied response to hypoglycemia, euthanasia induced by an overdose of general anesthetic at the end of the experiment was preceded by a generalized increase of electrical activity in virtually all recorded units (cells) of the adrenal medulla (**Figure 7A**) until activity abruptly halted. Some cells attained firing rates greater than 20 Hz for several seconds. In contrast to the stress response evoked by low glucose levels, there was no correlation between the initial and final firing rate (**Figure 7B**). Denervation almost completely prevented euthanasia-associated increase in firing (**Figure 7C**). These data demonstrate that that the increase in unit firing upon euthanasia is driven by neuronal input from the splanchnic nerve, and not by autonomous chromaffin cell activation.

## Discussion

We have adapted extracellular multi-electrode array technology, which had been developed to probe neuronal networks in the central nervous system, to detect adrenal chromaffin cell activity in living anesthetized rats. This approach enabled us to identify and track the electrical activity of individual chromaffin cells in the living anesthetized animal. To our knowledge, this is the first application of the technology to an endocrine tissue. As discussed below, the methodology was successful in recording from as many as 40 cells simultaneously for tens of minutes in animals under basal conditions and during stimulation of the splanchnic nerve. The study revealed independent networks of chromaffin cells that had varied responses to stress, suggesting that chromaffin cells *in vivo*, rather than acting homogenously, have nuanced responses to central nervous system activation.

### Chromaffin Cell Activity in Living, Anesthetized Rats Is Primarily Driven by Splanchnic Nerve Stimulation

An important finding of the present study is the primacy of neuronal stimulation, rather than pacemaker and spontaneous depolarizations, in causing action potentials in chromaffin cells in intact adrenal medulla in living animals. Large extracellular potentials (greater than 50 μV) that reflect currents generated by cellular action potentials almost completely disappeared upon interruption of splanchnic nerve activity by application of tetrodotoxin to the nerve or upon severing the nerve. The relatively rare action potentials detected after halting splanchnic nerve activity may reflect intrinsic action potential activity (22). The primacy of neuronal stimulation was also suggested in a study that recorded intracellular action potentials in freshly removed and bisected guinea pig adrenal glands (26) and in a study that detected catecholamine secretion using cyclic voltammetry and amperometry in rat adrenal slices (9). However, the conclusion seems to contradict studies in isolated chromaffin cells and in adrenal gland slices that detected non-neuronally evoked action potentials resulting from pacemaker activity (27, 28). Indeed, a large body of work has documented an array of voltage-sensitive Ca^2+^ channels and voltage and/or Ca^2+^-sensitive K^+^ channels in chromaffin cells, some of which underlie the observed pacemaker activity (see reviews (22, 29). We suggest that the state of chromaffin cells in the adrenal gland in the living adult animal with intact innervation and an intact, nourishing blood supply suppresses pacemaker activity. It should be noted, however, that chromaffin cells are not significantly innervated in newborn rats (30). Hypoxia causes catecholamine secretion (31) not through neuronal stimulation but through regulation of hypoxia-sensitive ion channels (29, 32).

What may be the function of the rich ‘palette’ of ion channels (22, 29) in adult chromaffin cells? Triggering of the action potential is only the first step in a cascade of events leading to exocytosis. Catecholamine secretion (measured by amperometry) can be closely coupled to the action potential (synchronous, within 25 ms) but can also occur 100’s of milliseconds (asynchronous) after the action potential when the membrane is partially repolarized (33, 34). The variety of voltage-sensitive Na^+^, Ca^2+^, and K^+^ channels determine ion flow during and after the action potential and likely regulates the next critical step, the rise of cytosolic Ca^2+^ at fusion sites. Thus, although the present study demonstrates the primary importance of splanchnic nerve in activating action potentials in adult chromaffin cells, the dynamic and regulated array of ion channels underlying the action potential in the chromaffin cell plasma membrane probably shapes the kinetics of Ca^2+^ influx and Ca^2+^- triggered exocytosis.

### Multicellular Chromaffin Cell Networks

The multielectrode arrays enabled the generation of a template of waveforms across multiple electrodes (16) in response to a single chromaffin cell action potential. These waveforms often contained highly temporally correlated contributions from other cells. The method had extraordinary temporal resolution with 30,000 measurements/sec. Coupled cellular responses separated by less than 100 μs to a few milliseconds were readily detected (**Figure 3**). The analysis required amassing thousands of events over time. The analysis revealed networks as large as 8 cells (**Figure 3**) that were stable for tens of minutes. The network sizes were likely underestimated because of the two-dimensional layout of the electrodes.

What accounts for the coupling? One possibility is electrical coupling through gap junctions. Gap junctions have been well documented in adrenal medullary slices (2, 3, 5, 6) and *in vivo* preparations (14, 35). Gap junctions complement synaptic transmission to increase catecholamine secretion (3). However, gap junction coupling likely accounts for only a small fraction of the action potential-coupled cells detected in the present study. Gap junctions of adrenal chromaffin cells are generally high resistance, and unable to conduct sufficient current between cells to transmit action potentials from one cell to another (coupling ratios significantly less than one) (2, 5). Furthermore, the latency of transmission of Ca^2+^ waves between coupled cells is 40 ms (5), much too slow to account for action potential latencies in the waveforms of milliseconds or less as detected in the present study. Although, gap junctions may be important for metabolic coupling and fine tuning of the secretory responses, they are unlikely to be the primary mechanism for action potential-coupling.

It is much more likely that the low latency coupling in a network is neuronal as has been previously suggested (36). Networks may reflect innervation of multiple cells by a single nerve fiber. Indeed, unmyelinated axons emerge from Schwann cell envelopment to innervate individual chromaffin cells. Neuronal coupling is suggested by electron microscope studies that show nerve terminal branching (37) and nerve endings making synapses with more than one cell (38). Light microscopy demonstrates nerve fibers with at least one and sometimes several boutons per chromaffin cell (36). The median distance and median latency between coupled cells were 95 μm and 0.59 ms, respectively (**Figure 4D**). By combining these two measurements, one estimates a conduction speed of 0.16 m/s, which is appropriate for small, unmyelinated axons (39–41). The fixed firing sequence in a network also supports the notion of propagation along nerve fibers and activation of postsynaptic chromaffin cells in a specific order. Coupling between neighboring cells sometimes had latencies of several milliseconds (**Figure 4D** **, left panel**), longer than would be expected by single fiber neuronal coupling. These longer latencies could reflect network innervation by two or more axons whose activities are coupled in the CNS.

It is attractive to suggest that coupled cells are defined by a cell cluster, the anatomical unit in the adrenal medulla (36, 42). Our findings suggest that this is not always the case. The diameter of histological cell clusters is approximated 100 μm (**Figures 1B, C** **)**. Forty-seven percent of coupled cells were within 100 μm of each other (**Figure 4D**); these pairs could reside in the same cluster. However, a significant fraction of synchronized cells (18%) were separated by greater than 200 μm and there were four cell pairs (6%) separated by greater than 400 μm. Furthermore, anatomical cell clusters likely do not limit coupling to one network since independent networks sometimes overlapped spatially (**Figure 3**). Thus, a chromaffin cell network may not be defined by a cell cluster, but rather by the temporal synchronization of a group of chromaffin cells regardless of their localization.

It should be noted that under chronic cold stress, gap junction electrical coupling becomes more robust (43) and could potentially contribute to action potential propagation between cells. These conditions were not explored in our study.

It is possible that different cell networks have different secretory outputs. Chromaffin cells contain and secrete epinephrine (the major catecholamine in the adrenal medulla) or norepinephrine depending on the cellular expression of the epinephrine synthesizing enzyme, phenylethanolamine-N-methyl transferase. There is evidence that different inputs from the CNS selectively cause epinephrine or norepinephrine secretion (44). Secretory granules (chromaffin granules) in chromaffin cells contain not only catecholamine but many peptides and hormones (for review (45)) including enkephalins (46), NPY, chromogranins, tissue plasminogen factor (tPA) (47) and plasminogen activator inhibitor-1 (PAI-1) (48). The mix of granule proteins differs from cell to cell (49–51). It is possible that one of the purposes of the specific network responses to stress (see below) is to modulate the cocktail of substances released into the blood during stress.

### Nuanced Response to Physiological Stress

The *in vivo* recordings allowed for the first time the simultaneous analysis of many individual cell responses to physiological stress. Rats were subjected to insulin shock-induced hypoglycemia. As expected, there was an increase in chromaffin cell firing rate coincident with the hypoglycemia as part of the stress response that seeks to maintain blood glucose. The response is driven by splanchnic nerve stimulation (23–25, 52, 53). Surprisingly, the increase did not occur uniformly (**Figure 6**). In some networks the increase was vigorous and sustained but in others it was short-lived or did not occur. In fact, networks that did and did not respond were sometimes spatially overlapping. These results suggest that there is a significant degree of information processing, probably in the central nervous system, that is responsible for the nuanced responses. Indeed, recent neuron tracing experiments indicate that there are extensive neuronal circuits originating in the brain stem and cerebral cortex whose final outputs are the adrenal medulla *via* the splanchnic nerve (17, 18). Electrophysiological studies also indicate discriminating responses emanating from the CNS (44). The nuanced response to metabolic stress contrasts with the response to stress induced by agonal respiratory arrest (**Figure 7**). In this case, there was a uniform increase in chromaffin cell firing rate followed by cessation, presumably because of central neuronal activation at onset of loss of CNS function. The notion that the stress response is varied and dependent upon the stimulus is consistent with previous analyses (54).

Under stress conditions our recordings occasionally showed recruitment of neighboring chromaffin cells into an active network. These changes may occur by different mechanisms including increased frequency of neuronal input and increased synaptic strength that brings previously silent cells to threshold (55), local changes in a cell’s electrophysiological properties induced by presynaptic factors (e.g. PACAP, peptides) (6, 56), or paracrine factors released from active chromaffin cells that modify cell-cell electrical coupling and network activity (57). Prolonged physiological stress may also lead to altering the constellation of ionic channels in chromaffin cells (22, 58).

This initial study with a multielectrode array in the adrenal gland *in vivo* raises many questions and future research opportunities. We did not delve deeply into the post-synaptic pharmacology of synaptic transmission. It is complex. However, we did investigate the role of nicotinic cholinergic receptors, the major rapid excitatory pathway in the adrenal medulla. The intraperitoneal administration of the nicotinic cholinergic antagonist, mecamylamine, inhibited transmission in approximately 50% of the cells. Muscarinic receptors also play a role in cholinergic stimulation of rat chromaffin cells but to a lesser degree than nicotinic receptors (59–61). Activation of these receptors by nerve stimulation may account for nicotinic antagonist-resistant action potentials. It is unlikely that pituitary adenylate cyclase-activating peptide (PACAP), which is present together with acetylcholine in splanchnic nerve terminals in the adrenal medulla, was responsible for action potentials upon nicotinic receptor blockade. Although PACAP release is essential for the strong secretory response to intense stress (58, 62–65), the cellular response to PACAP is through GTP-coupled receptor activated pathway that can take seconds to be manifest (65) and alone does not stimulate action potentials (58). Thus, the inability of the nicotinic antagonist mecamylamine to block synaptic transmission of some cells suggests that the pharmacology of synaptic transmission is not uniform between chromaffin cells (66). Indeed, the multi-electrode array and signal processing approach could be used in the future to decipher the ion channel code that regulates the electrophysiological and secretory output of the adrenal medulla in living animals during basal and stress conditions.

The adrenal medulla and islets are both endocrine tissues. However, there are striking differences in the functional organization of chromaffin cells in the adrenal medulla and insulin-secreting β-cells in pancreatic islets. The main signal for secretion in the adrenal medulla is neuronal through splanchnic nerve preganglionic cholinergic innervation that activates nicotinic ionic channels on chromaffin cells. The present study demonstrates that chromaffin cells are organized in small cellular networks that are coupled mainly through neuronal innervation with latencies between cells of less than a millisecond to several milliseconds. In contrast, the main stimulus for insulin secretion is the extracellular glucose concentration, which indirectly increases cytosolic Ca^2+^. β-Cell responses are coupled through the extracellular glucose concentration and gap junctions between cells with latencies of many seconds (67–70). Post-ganglionic stimulation by sympathetic and parasympathetic nerves modulates the response to glucose through relatively slow acting GTP-coupled receptors.

Finally, this *in vivo* study was performed on isoflurane anesthetized rats, and it is possible that the stress response we measured was reduced by the anesthesia (71). This limitation will be overcome in the future as techniques are developed to explore adrenal function in freely behaving animals (72).

## Data Availability Statement

The raw data supporting the conclusions of this article will be made available by the authors, without undue reservation.

## Ethics Statement

The animal study was reviewed and approved by Institutional Animal Care & Use Committee (IACUC) at University of Michigan.

## Author Contributions

Original idea by RH and ES. Experimental design by JL, SE, RH, and ES. JL carried out the experiments and data analysis. The manuscript was written and reviewed by JL, SE, RH, and ES. All authors contributed to the article and approved the submitted version.

## Conflict of Interest

The authors declare that the research was conducted in the absence of any commercial or financial relationships that could be construed as a potential conflict of interest.

## Publisher’s Note

All claims expressed in this article are solely those of the authors and do not necessarily represent those of their affiliated organizations, or those of the publisher, the editors and the reviewers. Any product that may be evaluated in this article, or claim that may be made by its manufacturer, is not guaranteed or endorsed by the publisher.

## Supplementary Material

Supplementary Figure 1 Age and gender related baseline firing rate metrics. A total of 21 females and 17 males were included in this analysis, with an average age in days of 77.33 ± 26.17 and 66.71 ± 31.58 respectively (ns, *p*=0.1626). Two females with an age of 250+ days were excluded from the analysis. The firing rate for each subject was calculated by averaging the baseline firing rate of all the recorded units in that experiment, cells with an 85+% of correlation were counted as a single unit (network of cells). **(A)**. There were no significant differences between baseline firing rates of females when compared to males (ns, *p*=0.6558) for the studied age range. **(B, C)**. The average firing rate in females and males display similar exponential decay when correlated with age (f(x) = a*exp(b*x), female: a= 2.89 b= -0.007393, male: a= 2.979 b= -0.008994).
