# Supplementary figures and images for: Basal and Stress-Induced Network Activity in the Adrenal Medulla In Vivo
Source: Front Endocrinol (Lausanne). 2022 Jun 20;13:875865. doi: 10.3389/fendo.2022.875865 (PMC9250985; doi:10.3389/fendo.2022.875865)

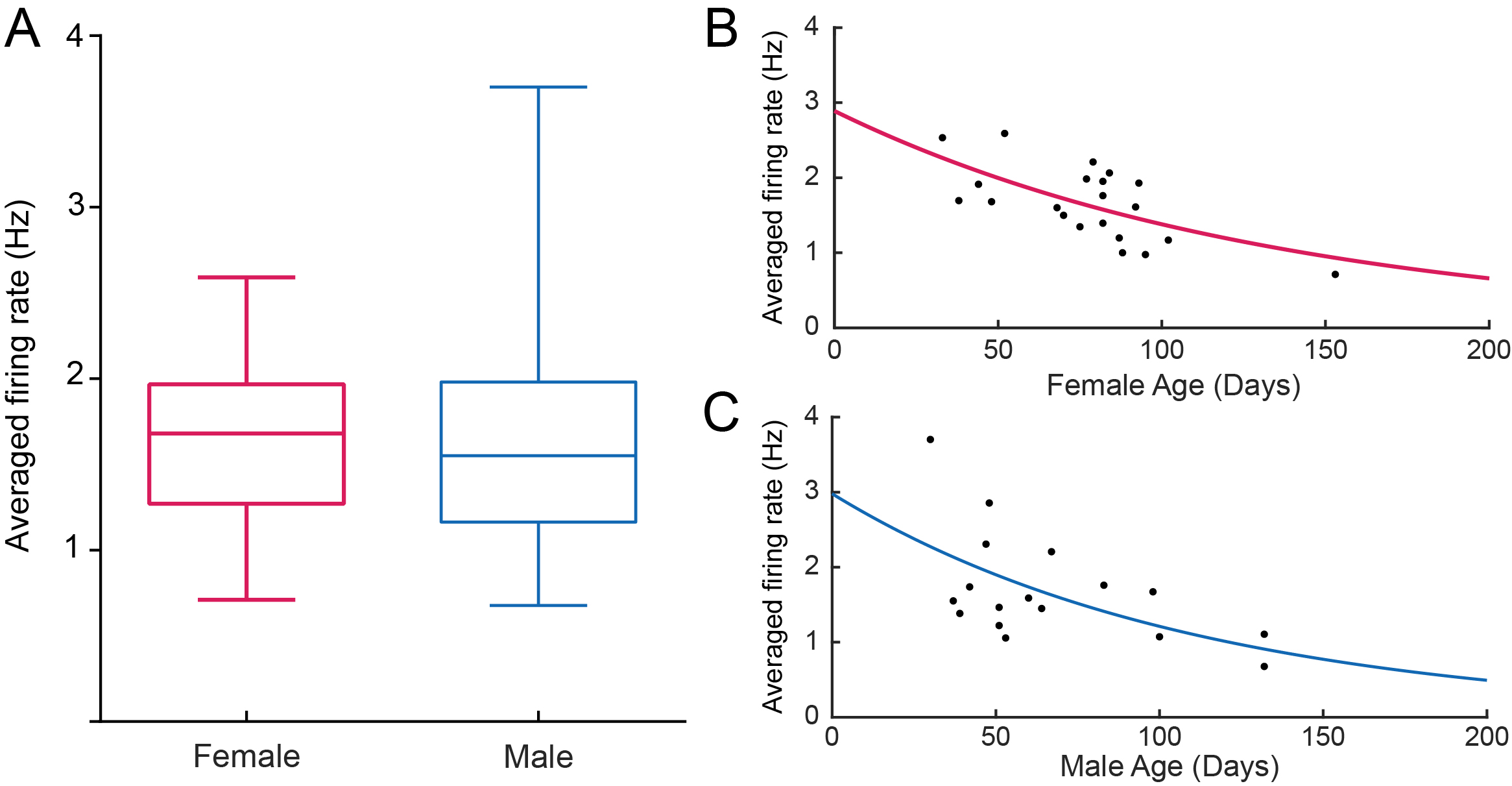

Supplement: Supplementary Figure 1 — Age and gender related baseline firing rate metrics. A total of 21 females and 17 males were included in this analysis, with an average age in days of 77.33 ± 26.17 and 66.71 ± 31.58 respectively (ns, p=0.1626). Two females with an age of 250+ days were excluded from the analysis. The firing rate for each subject was calculated by averaging the baseline firing rate of all the recorded units in that experiment, cells with an 85+% of correlation were counted as a single unit (network of cells). (A). There were no significant differences between baseline firing rates of females when compared to males (ns, p=0.6558) for the studied age range. (B, C). The average firing rate in females and males display similar exponential decay when correlated with age (f(x) = a*exp(b*x), female: a= 2.89 b= -0.007393, male: a= 2.979 b= -0.008994). [file Image_1.jpeg]
